# Veganuary and the vegan sausage (t)rolls: conflict and commercial engagement in online climate-diet discourse

**DOI:** 10.1057/s41599-022-01464-2

**Published:** 2022-12-19

**Authors:** Mary Sanford, Jamie Lorimer

**Affiliations:** 1 Oxford Internet Institute, University of Oxford, Oxford, UK; 2 School of Geography and the Environment, University of Oxford, Oxford, UK

**Keywords:** Cultural and media studies, Business and management, Politics and international relations

## Abstract

Social media platforms have become critical venues for a wide spectrum of influence campaigns, from activism to advertising. Sometimes these two ends overlap and it remains unknown how the latter might impact the former. Situated within contemporary scholarship on vegan activism, this work examines corporate involvement with the Veganuary 2019 campaign on Twitter, as well as the antagonistic backlash it received. We find that the activists and commercial entities engage mostly separate audiences, suggesting that commercial campaigns do little to drive interactions with Veganuary activism. We also discover strong threads of antagonism reflecting the “culture wars" surrounding discussions of veganism and climate-diet science. These findings inform our understanding of the challenges facing climate-diet discourses on social media and motivate further research into the role of commercial agents in online activism.

## The Greggs Vegan Sausage Roll incident

**Fig. 1:**
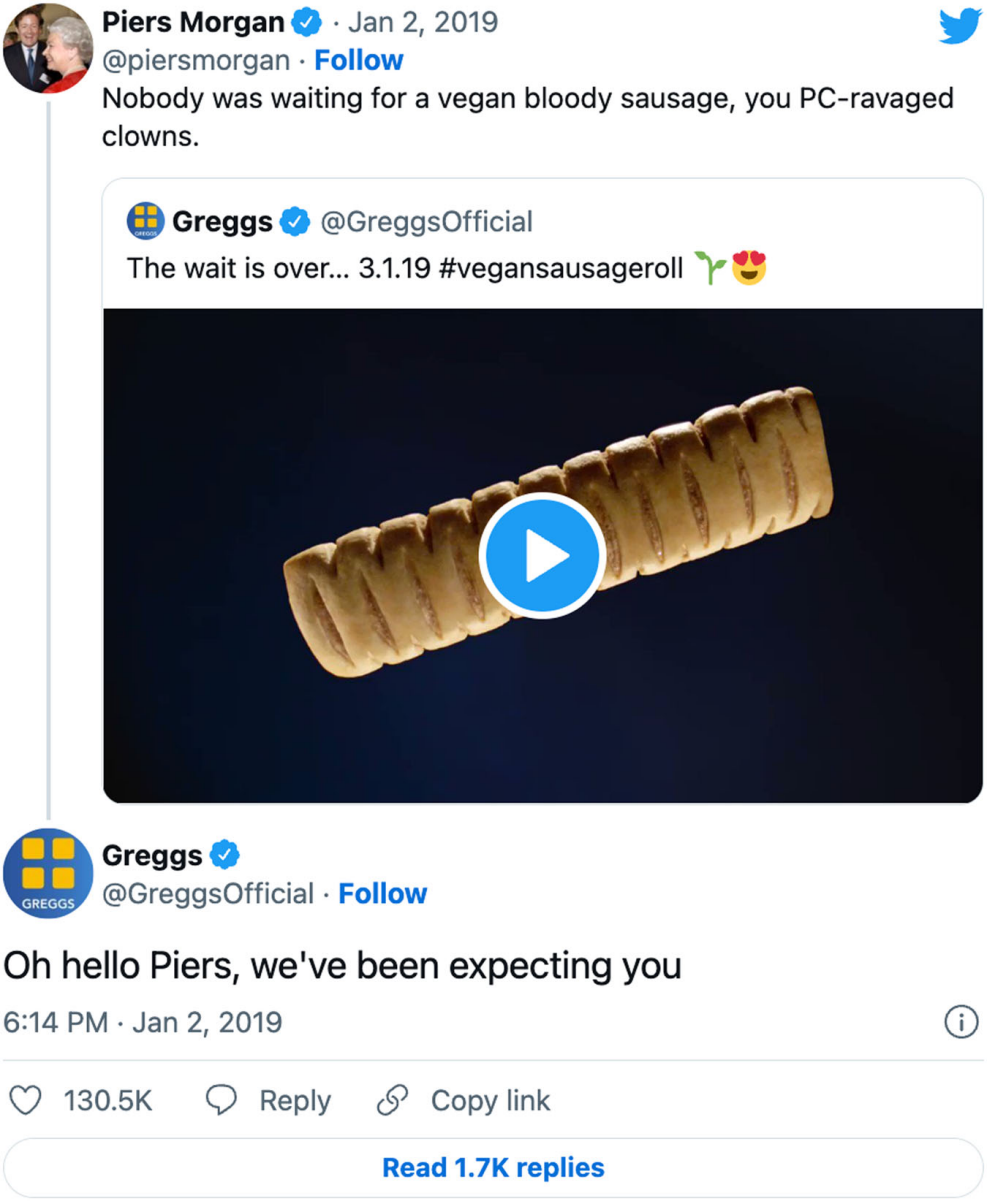
Initial tweet exchange between Piers Morgan and Greggs. A screenshot, taken by the authors, of the post in which Greggs announced the launch of the vegan sausage roll with Morgan’s initial reply and Greggs’ counter below.

On Jan 2 2019, UK fast-food giant Greggs announced the launch of a new vegan version of their traditional sausage roll on the social media platform Twitter. The vegan sausage roll had been specially made by Greggs to coincide with Veganuary—the month-long campaign led by the vegan organisation with the same name to encourage people to go vegan for January. The popular, right-wing TV presenter Piers Morgan responded to the launch with a string of disdainful tweets. The savvy marketing team at Greggs promptly responded, seeking to make the best of Morgan’s tirade (Fig. 1). A few days later, Morgan ate a vegan sausage roll live on *Good Morning Britain*, promptly spat it out, labelled it disgusting, and returned to Twitter to further lambast Greggs. Discussion subsequently escalated, going viral to draw in a large, diverse and often antagonistic range of social media users who debated the ethics and politics of vegans, veganism, meat and corporate involvement in food politics. Despite Morgan’s counter-campaign, the release of the vegan sausage roll boosted Greggs’ sales by 58% in the first half of 2019 (Starostinetskaya, 2019).

In this paper, we take Veganuary and the Greggs Vegan Sausage Roll (GVSR) incident as illustrative of two important dimensions of contemporary food and cultural politics as they play out on social media. The first is the growing corporate involvement in social media activism, in which prominent brands seek to align themselves with good causes in the interests of brand enhancement and driving sales. The second is the often antagonistic backlash to this involvement from both the political left and the political right that manifest in what have become known as online ‘culture wars’. This paper offers an in-depth analysis of the online community and the social media discourse associated with Veganuary 2019, focusing in particular on the GVSR in order to address two broad aims: (i) to examine the character and the effectiveness of corporate involvement in veganism and; (ii) to describe and explain the content and sentiment of the vegan culture wars.

In so doing, this paper offers an analysis of the users, narratives, and communities of the Veganuary 2019 discourse, contextualised within literatures examining contemporary veganism and controversy in climate-diet discourses. While previous studies have begun to examine discussions of veganism on social media and in mainstream media (Cole & Morgan, 2011; Goodman & Jaworska, 2020; Morris, 2018), we offer the first examination of the structural implications of commercial engagement with veganism and climate-diet discourse and an evaluation of its potential to serve as a bridge between commercial audiences and activism. Our findings inform current discussions regarding the state of climate-diet discourses on social media and fix a spotlight on the role of commercial agents in online activism. We identify powerful streams of politicised antagonism to Veganuary activism, some actually fuelled by commercial engagement, and examine the ways in which social and political tensions co-opted the discourse. We show how veganism has become exemplary of the hot topics that serve as performative flashpoints for the online expression of entrenched and antagonistic opinions, splitting those on the left and the right of British politics.

## Veganism goes mainstream, with a side-serving of backlash

Veganism has been popularised in Western contexts in recent years as an effective measure for reducing environmental harm, especially by tackling the carbon emissions and land use changes associated with livestock, agriculture, and meat- and dairy-based diets (Godfray et al., 2018; IPCC, 2019; Poore & Nemecek, 2018; Willett et al., 2019). There is low but growing public awareness about the implications of consumption and dietary choices on the environment (Kristiansen et al., 2020; Neff et al., 2009; Pendergrast, 2016) and more consumers are seeking vegan alternatives, if not fully vegan lifestyles (Shoup, 2019). Some argue that veganism has become ’cool’, appealing to younger, urban, and wealthier demographics (Doyle, 2016; Jallinoja et al., 2018; Nguyen, 2017). This mainstreaming of veganism has created new ways of understanding it “as a tripartite practice of health, animals, and the environment," in comparison with earlier conceptions of the movement as radical or extreme (Oliver, 2021, p. 209). It has also presented economic opportunities for corporations seeking to capitalise on this new consumer segment, fuelling innovation and growth in sales of novel ‘plant-based’ meat and dairy alternatives (Santo et al., 2020). Companies in this sector often present these products as animal-saving, environment-healing, and social justice-promoting choices (van der Weele et al., 2019).

This shift has been driven by, and now empowers, online activist movements such as Veganuary who use social media campaigns to spread awareness and to encourage people to try veganism. Since it launched in 2014, a central aim of Veganuary has been to collaborate with corporations to "make plant-based foods more visible, tasty and accessible to the population," seeing this as an important means of “break[ing] down the main barriers to vegan eating" (Veganuary, 2022a, p. 4). This framing encourages flexible and accessible 'middle-ground' solutions (Jallinoja et al., 2018) over strict adherence to stringent dietary regimes. Both on social media and on the organisation’s official website, Veganuary advocates use terms such as ‘plant-based’ and ‘flexitarian’ as part of a strategy to make the vegan lifestyle seem more approachable and less extreme, to the benefit of corporations wanting to expand their businesses and to activists hoping to encourage hesitant meat consumers to consider alternatives (Veganuary, 2022b).

This emerging model of mainstream, corporate or ‘Big Veganism’ (Sexton, 2018) has been criticised on several fronts. First, by ‘traditional’ vegan activists and other advocates of alternative food networks, who are generally on the political left. While some vegan activists (such as those at the People for the Ethical Treatment for Animals) are happy with a reduction in meat consumption by whatever political and economic means (Dutkiewicz & Dickstein, 2021), traditional vegan critics suggest that merely promoting and selling vegan products is not enough. They argue that more must be done to educate consumers about the problems with meat and dairy and to change the status quo. They suggest that Big Veganism is ineffective or worse, sheer capitalist opportunism (Guthman, 2008; White, 2018) and caution that it will lull consumers into a false sense of righteousness, preventing critical evaluation and widespread change (Wrenn, 2011).

Anarchist vegan scholar Richard White (2018) explains how the older model of veganism was "never just about food choices but is rather a radical activist praxis (in both theory and action) of a manifest desire to act in a way that prefigures an interspecies politics of justice and total liberation" (p. 5). He sees recent Big Veganism as completely divorced from these principles, and therefore diluting the collective understanding of what it means to be vegan. Giraud (2021) summarises the concerns of many vegan scholars and activists that the popularisation of vegan foods leads to the loss of "the more radical dimensions of veganism that characterised its origins" in favour of veganism as purely a dietary choice, without the "holistic understanding of vegan practice that questions human-animal relations more broadly and their connection with other forms of oppression" (p. 8).

These critics argue that Big Veganism offers a ‘palatable disruption’ (Clay et al., 2020); a change to the food system that does not actually disrupt its underlying values and power dynamics. As such it exemplifies the neoliberal political orthodoxy in which consumer choices are framed as the key lever to regulate harmful corporate activity. Food justice advocates argue that the growing industry of meat-free, plant-based alternatives merely secures the political economy of the mainstream corporate food system that was once the target of vegan critique (Butcher, 2018; Dominick, 2015; Guthman, 2008; Harper, 2010; Twine, 2012; White, 2018; Zarling, 2018). These debates have been extensively covered in academic literature (see Giraud (2021); Santo et al. (2020); Sexton et al. (2022) for a comprehensive review).

The rise of Big Veganism has also been criticised by some representatives of the mainstream meat and dairy industries, as well as by a heterogeneous collection of pro-meat and anti-environmentalist voices on the political right. Some of these argue that livestock can and should play a vital role in sustainable landscape management, and that meat and dairy are a necessary part of a healthy diet. They promote either a ‘less and better’ model of ‘regenerative agriculture’ that would support small-scale traditional farming and shift towards high quality and low-intensity production (Fairlie, 2010; Giller et al., 2021; Percival, 2022). Or they advocate for a ‘more, but greener’ model of sustainable intensification involving the scaling up of production to increase yields, and the further application of biotechnology for mitigating emissions and the harms associated with land use change (Godfray & Garnett, 2014). Both approaches make strong appeals to a pastoral ideal of livestock production as a traditional and significant part of European and American culture (Monbiot, 2022). For an overview of these arguments and the debates they provoke, see Cusworth et al. (2022) and McGregor & Houston (2018).

Existing work has shown how online discussion and controversy about the merits of veganism peaks in response to the publication of high-profile reports. For example, parallel to the Veganuary 2019 discourse, online debate focused intensively on the EAT-*Lancet* report, which was published in early January 2019 and examined the negative consequences of the global food system’s reliance on meat and the benefits of a plant-based diet (Willett et al., 2019). This report precipitated an intense and discordant debate and led to the emergence of the hashtag *#yes2meat*, which became a focal point for the pro-meat discourse (Garcia et al., 2019). Similarly, in August 2019 the Intergovernmental Panel on Climate Change reported on the detrimental environmental impact of the livestock industry (IPCC, 2019). Although the report made no explicit recommendation for diet change, the ensuing discourse on Twitter was heavily polarised and contained high levels of toxicity, i.e., disrespectful, rude, or otherwise unreasonable comments (Sanford et al., 2021). Moreover, Olausson (2018) and Olausson (2019) find significant polarisation in discussions pertaining to meat versus vegan diets and the science advocating the latter as climate action among Swedish Facebook users. These studies demonstrate the polarisation and conflict in climate-diet discourses, specifically those related to the science underlying the link between the meat industry and climate change. However, they have hitherto focused on discourses anchored in discussions of scientific reports and have yet to examine fraught contexts in which commercial engagement targets individual consumption choices, such as with Veganuary.

### A note on terminology

There is strong debate amongst scholars on how researchers should define veganism, from those favouring a simple practice-only definition (Dutkiewicz & Dickstein, 2021) to others advocating a more holistic praxis-based conception (White, 2018). Without wanting to oversimplify the concept, we adopt a definition of veganism, which best resembles what the Veganuary organisation advocates: the practice of abstaining from animal-based products for the purposes of protecting animals, the environment, and improving one’s health. Throughout the paper, we use the terms 'vegan activism' or 'vegan activists' to refer to anyone advocating for veganism within the Veganuary context. We do not intend to generalise beyond the movement as the full scope of vegan activism covers a highly complex tapestry of beliefs, motivations, and practices.

## Data and methods

This paper endeavours to examine the character and effectiveness of corporate involvement in veganism, as well as to describe and explain the content and the sentiment of the vegan culture wars. To deliver on these aims we use a network approach to identify the structures of influence and audience interaction in the discussion of Veganuary 2019 on Twitter. Thinking with networks offers an established way of understanding the social, and a helpful spatial metaphor for theorising the place and role of social media in contemporary society. Our approach builds on rich literatures in sociology, which underscore the importance of social ties in influencing opinions, intentions, and behaviour, beginning in earnest with Granovetter (1973), and further literatures, which trace the ways in which the social is materialised and performed through digital media (Castells, 2009; Taffel, 2019). It also directly responds to the call made by Yang & Saffer (2019) to use network science and theory to "examine how digital networks of activists form online and how such networks interact with other social actors... [and] the connection between polarising ideas and discourses” (p. 6).

Here, we focus on the digital discourses in any and all messages posted or exchanged between users pertaining to Veganuary on Twitter in January 2019. We use the term *discourse* in a Foucauldian sense to describe the power of text to frame, define, normalise and police everyday behaviour. We are interested in how the practices described and contested on social media enact forms of food system governmentality with real world consequences for what people eat and what people associate with acts of eating (Sexton, 2018).

We chose Twitter because it was the main platform used by the Veganuary organisation for the 2019 campaign. Social media platforms such as Twitter have become a critical arena for information spreading, public debate, and opinion formation (Fuchs, 2021), challenging traditional news media as sources of information among certain demographics (Digital News Report, 2021; Walker & Matsa, 2021). A broad literature demonstrates how social media has become increasingly influential as a means of mobilising protest action, including for environmental issues (Anderson, 2017; Askanius & Uldam, 2011; Boykoff & O’Neill, 2011; Chen et al., 2022; De-Lara et al., 2022; Hopke & Hestres, 2018; Molder et al., 2022). We examine the Veganuary discourse on Twitter as a potential space for not only encouraging vegan consumption habits but also as an example of what Stolle & Micheletti (2013) refer to as "discursive political consumerism" —using communication and deliberation to change how people view consumption and how corporations assess social responsibility (p. 171). We consider the messages posted on Twitter by the Veganuary organisation as representing a snapshot of the organisation’s attempts to inform, educate, and engage their public about why veganism is important. At the same time, Twitter users engaged with this content and its supporters, along with the content posted by commercial entities, via the action of sharing select content to their own profiles (known as a ‘retweet’). These interactions spread the shared information to new audiences, thereby constructing a discursive environment pertaining to Veganuary, which can be represented as a network.

We collected a sample of the Veganuary discourse using Twitter’s search API, as it functioned in 2019. At the time, the API took search queries, i.e., keywords, and returned a random sample of all tweets within the last seven days matching those keywords. Our dataset was built by querying the API with the keywords *vegan, veganuary*, and *veganuary2019* four times during the month of January 2019. We use these terms as they were identified as the key hashtags used by the official Veganuary account in their posts. The results of each query were combined and duplicates removed. The final dataset contains over 460k tweets. As Veganuary is a UK-based organisation, the majority of tweets are written in English and are from UK users, but there are also tweets from the US, Australia, New Zealand, Canada, Turkey, Sweden, Italy, Germany, France, and Japan.

The data collection and ensuing methodological pipeline were approved by the University of Oxford’s Social Sciences and Humanities Interdivisional Research Ethics Committee, but we would like to highlight a few key considerations. While Twitter only allows collection of tweets from public profiles, none of these users explicitly consented to having their tweets analysed for the specific academic research purpose of this paper. Instead, the users agree to Twitter’s general terms and conditions when they join the platform, and these include any academic research purpose the platform deems fit to permit. While this mode of granting permission has been construed as potentially ‘paternalistic’ and therefore unethical (von Benzon, 2019), it is standard practice for researchers to protect the anonymity of users in the Twitter samples they collect, with the exception of public figures and organisational accounts, following the framework set out by Williams et al. (2017). This includes refraining from publishing the names and direct tweet quotations of individual accounts. In this work, we adhere to these rules and only directly name and/or quote public figures, corporations, and organisations. Moreover, in the results section, we include examples of tweets from individual accounts, which have been paraphrased so that they may not be searched and identified.

### Identifying discourse structure

To identify the dominant interactions and communities in the Veganuary discourse we used a retweet network. We choose to work with retweets because researchers have determined that of the modes of interaction on Twitter, including likes, mentions, followers, and retweets, the latter are the most reliable signal of influence spread (Cha et al., 2010; Kwak et al., 2010). Likes can also constitute a form of influence, as they indicate appreciation and signal agreement with the content conveyed in the tweet (Lipsman et al., 2012). However, it is not possible to see who has liked a given post at scale, and therefore it is not possible to construct a network of users based on this mode of interaction. Reply and mention interactions are traceable but have been determined to not bear the same intent nor information content as retweets, i.e., mentions and replies are mostly meant to engage others in conversation instead of spreading information (Cha et al., 2010; Kwak et al., 2010). They are therefore considered to have lower influence potential. See Section 1 of the Supplementary Materials (SM) for further discussion of this point.

This approach enables us to identify how the discourse environment was shaped by the activity of key stakeholders (Veganuary activists and commercial entities), specifically how these users influence their audiences, and how this activity builds links between commercial audiences and Veganuary activists. This type of influence on Twitter is distinct from the object of other kinds of influence analyses, e.g., Goodman & Jaworska (2020), which compare the ‘influence stature’ of key influencers based on the size of their digital audience. Instead, our approach is inspired by the work of Becatti et al. (2019), who used the retweet network to extract the community structure of the target discourse, i.e., groups of users who engage with each other more than those outside their group, and thereafter signals of influence spread within it. With careful annotation, the retweet network allows us to characterise the identities and narratives of each community, as well as any conflict or controversy between them. The community structure then provides us with the input we need to determine how the audiences of the communities overlap.

There are many different ways to construct a retweet network. Some researchers use directed networks with edges weighted by the number of times one user retweets another (Cherepnalkoski & Mozetič, 2016), some with unweighted edges (Grčar et al., 2017), while others use undirected weighted edges (Evkoski et al., 2020). In this work, we use the latter and define the retweet network by connecting user A to user B if user A has retweeted a tweet originally posted by user B. This connection, or edge, is weighted by the number of times user A retweeted user B and is undirected. In the case that users A and B retweet each other, the edges are summed. See Section 1 of the SM for further justification for using undirected over directed edges in our case.

In our construction, we exclude the first-level network *leaves*, i.e., the users in the network that only connect to one other user. These users interact just once with the discourse and as such their engagement is not viewed as significantly active or impactful, therefore removing them helps more meaningful interactions to stand out (Mastroeni et al., 2020; Stewart et al., 2018). The resulting network yields an overview of who retweeted whom within the sample, and with what frequency. As we are interested in identifying the community structure of the network, a community detection analysis using the Louvain algorithm is implemented (Blondel et al., 2008). Based on this community detection, we calculate the modularity and assortativity of the network. Modularity provides an indication of how polarised the communities in the network are and assortativity quantifies the extent to which nodes preferentially attach to nodes similar to them. Further explanation of the algorithms, robustness checks, and definitions of modularity and assortativity are provided in Sections 2.1 and 2.2 of the SM.

### Labelling communities

To determine the themes and narratives active in each community, random samples of 200 tweets were examined for each community containing more than 1% of total retweets. We chose this threshold after discovering that communities smaller than this tended to be much more incoherent and random than the larger communities. It has also been used in previous work examining retweet networks (Stewart et al., 2018). In the first instance, the tweets were coded generally as pro-Veganuary, anti-Veganuary, and/or representing commercial engagement. We then refined these labels based on the prevailing narratives observed in the samples. For example, we differentiate commercial engagement that is explicitly tied to the Veganuary campaign from more generic engagement promoting vegan products without mentions of Veganuary. While we observe a range of different types of vegan activism in the sample, reflecting the diversity of types of vegan activism offline, we find that they are all concentrated in a single community. Therefore, we do not attempt to further classify the strands of activism as we are more interested in identifying how communities of significantly different ideologies and/or intentions interact in the network. Moreover, we observe a tremendous volume of content focused on the GVSR. Some of these posts are derogatory in nature, while others support the initiative. We categorise such content separately to capture this difference. Finally, we also find a variety of anti-Veganuary narratives and give them each their own label accordingly.

Once the labelling framework was finalised, we began the annotations. For each tweet, we manually recorded which label best applies. Once all tweets per community have been labelled, we counted how many times each label occurs in the sample for each community. A community is then given a name representing the label with the highest score, i.e., the one that is most frequent among the sampled tweets of that community. Section 2.3 of the SM contains further details of the annotation process, along with Table S1, which presents the annotation counts per community.

### Detecting audience structure

The retweet network analysis allows us to identify the discourse stakeholders, their communities and the narratives they propagated, as well as any polarisation or conflict between the communities. The second part of the analysis builds on the initial discourse mapping to uncover the extent to which the audiences consuming information via retweet in the network overlap, an indicator of reciprocal engagement, specifically between the activist and commercial communities. This analysis indicates how much the posts of the users in these communities were consumed by the same sets of users. Observing strong overlap between the audiences would constitute evidence that the audiences of the two communities are unified, i.e., that the commercial audiences also engaged with the Veganuary activists. If not, it would suggest that commercial engagement failed to drive its audience to engage with the activist core of the Veganuary campaign.

In order to measure this, we implement a network projection analysis inspired by Becatti et al. (2019). That work presents a bipartite projection model for extracting influence dynamics of the political discourse on Twitter leading up to the 2018 general elections in Italy. They use the method to identify signals of influence and political alliances between sets of politicians indicated by their shared audiences using a framework established by Saracco et al. (2015, 2017). While we are interested in identifying these ideological fault lines in the Veganuary case, we place higher priority on comparing the ways in which interactions may or may not spread from commercial engagement to other parts of the discourse. This is what the projection network accomplishes beyond the retweet network and why it is an appropriate method to use for our research objective.

The projection is built by first constructing a bipartite network between the most retweeted users, i.e., the primary content producers, from each community and identifying the full set of users who retweeted them, i.e., the content consumers. Then, this bipartite network is projected onto the producer users layer revealing a monopartite network connecting all users who were retweeted by the same sets of consuming users. Performing community detection on the monopartite network reveals communities of users with the most overlapping sets of consuming users. We then categorised the composition of these communities in terms of the retweet network communities, allowing us to determine which of the latter have the strongest tendency to have overlapping audiences. All necessary definitions and steps taken to build and validate the projection network are presented in Section 3 of the SM.

## Results

### Polarisation and contention in the discourse

Table 1 provides the name and descriptions of each of the largest twelve communities, including the proportion of the dataset they comprise in terms of retweets and users.

**Table 1:** Descriptive table of the top 12 communities in the retweet network.

Name	Description	Users	Retweets
GVSR–	Mixture of GVSR content with anti-vegan jokes and criticism of consumer activism	0.15	0.14
Core support	Veganuary-specific advocacy and engagement	0.14	0.22
GVSR+	GVSR commentary and other plant-based options in big food chains	0.14	0.12
Access/Trolls	General mixture of jokes and hate aimed at vegans, particularly for perceived elitism. Some vegan defence and aggression in return.	0.10	0.18
Mixed	Mixture of vegan activism (19%), Veganuary commercial promotions (16%), vegan defence (14.5%), aggression towards non-vegans and critics (9%), with many random posts (22.5%) tangentially mentioning veganism	0.10	0.09
Piers Morgan	Attacks on Veganuary and GVSR led by Piers Morgan	0.07	0.09
VPromo1	Vegan bloggers and influencers	0.04	0.03
News	Reporting on topics related to Veganuary: GVSR controversy, the carbon footprint of vegan vs meat diets, questioning the sustainability of vegan diets, recipes and guides	0.03	0.04
Conspiracies	Claims of corruption and conspiracy behind veganism and climate-diet science	0.03	0.02
Promo1	Brands and bloggers promoting vegan products	0.03	0.02
Promo2	Mixture of vegan recipe sharing and product promotion, little specific to Veganuary	0.01	0.01
VPromo2	Restaurants and brands promoting vegan specials for Veganuary	0.01	0.01

Includes the name given to each community, a qualitative description, proportion of unique users in the network, and proportion of number of the total volume of retweets received by users in the community.

The discourse breaks down into communities comprising four main themes: Veganuary activism, antagonism, commercial engagement, and GVSR discussion. The *Core Support* community comprises the bulk of activists in the discourse. It includes the official Veganuary account, regional PETA accounts and other prominent animal charities, and other vegan advocates, most of whom are normal individual users (i.e., non-public figures). The main objective of the posts in this community is to open up conversation about veganism with the greater Twitter user base and raise awareness about the movement, e.g.,

*Why did you go #vegan? Respond in the comments and retweet. #veganism #veganhour #VeganTuesday #HealthyLiving*

*Thank you to all who have committed to #Veganuary We all have our own journeys but if you want to hear some of ours, tune into our podcast #itallveganwithfriends where we hope to inspire you to go further and most importantly to not stop being #vegan*

Some users post about the benefits of going vegan, along with their favourite vegan products, recipes, and restaurants. For example,

*HEALTHY VEGAN SHOPPING LIST: for anyone who’s just starting a vegan journey or needs grocery inspo! Hope this helps*

*If you want a simple and easy to read guide to veganism then check this out! Happy veganuary!*

Others focus on emphasising the cruelty of the meat industry, for animals and the environment, along with the detrimental side effects on personal health, e.g.,

*Going vegan has only positive outcomes! Good for you, good for animals, good for the planet! #vegan*

There are also tweets defending Veganuary and its supporters, refuting challenges and criticisms, e.g.,

*‘I hate vegans’ means ‘I hate being reminded of the torture, exploitation, & murder that I facilitate when I eat meat’*

*‘I’d go vegan but it’s too expensive’ says someone who spends $5 on a latte & buys meat fast food weekly.*

There is also a small but noticeable volume of attacks against non-vegans and vegan critics for not doing enough to save the planet or to protect animals. There is even evidence of vegan infighting, i.e., certain vegans claiming that other vegans are not ‘true vegans’ if they are not vegan for the right reasons. This echoes Neuman (2020)’s studies of collective boundary building between different understandings of vegetarianism and veganism, as well as Greenebaum (2012)’s study of vegan identity and authenticity, specifically the tendency of ethical vegans to differentiate themselves from health and environmental vegans, who they see as being 'not sufficiently vegan.' For example,

*If you didn’t go vegan to save animals, you aren’t vegan, you’re plant-based. Change my mind.*

*ok idc [I don’t care] if this is controversial but veganism is a lifestyle, not a diet! if you’re not vegan in all your choices, then you’re not really vegan.*

The Veganuary-related promotional communities (*VPromo*) include influencers, bloggers, and several brands sharing recipes and promotions for vegan products (ranging from food, clothes, cleaning supplies, and cosmetics) via competitions and giveaways. For example,

*Oh, have we got a #veganuary deal for you! Just follow and RT to WIN four tubs of our delicious #dairyfree fudge! We’ll pick the winner on Friday. GO! #FreeFudgeFriday*

These VPromo communities differ from those labelled simply *Promo* as the latter are not tied to Veganuary, but constitute more generic promotion of vegan products, which could happen at any time of year, e.g.,

*#Vegamaro is the 1st #vegan #negroamaro #wine in the world. #FeudidiGuagnano*

There is a much wider variety of communities antagonistic to Veganuary in the discourse. The largest antagonist community, *Access/Trolls*, includes criticism and debate about the elitism and inaccessibility of vegan diets. This speaks to what Goodman & Jaworska (2020) find in their study of “good food” influencers on Twitter—many of whom promote vegan or otherwise plant-based diets—specifically with respect to how these influencers reinforce the image of veganism and other “good food” diets as privileged to the white, heteronormative, and middle- and upper-classes. It also includes a significant volume of general insults targeted at vegans. For example,

*Stop arguing about veganism in my mentions pls until a whole vegan meal can be cheaper than fast food don’t pressure people with low incomes to go full vegan that’s it that’s my point that’s all*

*You feel better eating vegan/vegetarian because you didn’t eat any vegetables before, Karen*

lol [laugh out loud] we don’t all want to be vegan and drink soy milk gosh please stfu [shut the f*ck up]

Some of the criticisms here also focus on the involvement of big corporations with the Veganuary campaign, and echo arguments made in the literature against ‘Big Veganism’, specifically about how it is not intrinsically free of exploitation, nor does it automatically guarantee a lower carbon footprint. For example, the following tweet captures this theme:

When corporations start paying works a proper wage and give them benefits then I’ll try f*cking veganism. There’s human exploitation and cruelty in veganism, prove me wrong

Another antagonist community, *Conspiracies*, focuses on contradicting the health and environmental benefits of vegan diets. Users in this community include a diverse range of nutritionists, food journalists, and far-right enthusiasts. They claim that veganism is part of a conspiracy led by left-wing politicians, large food corporations, and scientists, and that a lot of it, especially commercial participation, is elitist and insincere. They also tie Veganuary to the 2019 EAT-Lancet report, which served as a key focus point for conspiracy theorists in the discourse. Critics in this group claim that the report is corrupt, and that ‘real’ science finds that a vegan diet is detrimental to both one’s personal health and the environment. Most of this kind of criticism appears to be levied at 'junk food vegan' products, i.e., highly processed products made from artificial ingredients to look and taste like meat. The examples below illustrate these points:

*More of the same vegan propaganda supported by big food companies, making low nutritional foods, and and big pharma, making bank from consequential declining health through usual propagandist @guardian never questioning the always bad science #yes2meat*

*Activists for animal rights have infiltrated environmental and nutrition science…all getting money from big food and vegan elite who prefer to blame cows then their own private jets and corporate empires for climate change and fast food for poor health*

This denial of climate-diet science and calling into question the intentions of its practitioners is reminiscent of previous studies that demonstrate how the popularisation of science can be weaponised to erode public faith in its findings (Gunnarsson & Elam, 2012).

The most striking antagonist community surrounds Piers Morgan and his attacks on Veganuary and the GVSR. Users in this community include UK Conservative party pundits, farmer unions, QAnon enthusiasts and Trump supporters, and the account for the Russian Embassy in the US. The main narratives of these users were initially made in response to the GVSR but expanded to include general criticism of vegans for their perceived hostility, radicalism, and hypocritical virtue signalling. Morgan’s right-wing political stance and his ridiculing of vegans as “snowflakes” and “PC-crazed” liberals, attracted several posts from right-wing politicians, journalists, and commentators who joined the discourse to criticise veganism for its association with left-wing politics. In addition, Morgan claimed that vegan diets are actually more harmful to the environment than meat-based diets, due to the plastic packaging vegan items require, and as such implies that the narrative of veganism for climate change is a hoax. The tweets below illustrate, the first is from Morgan himself:

*I only moan about PC-crazed, gender-fluid obsessed, radical vegan/feminist snowflakes slowly wrecking the Planet. Whilst eating obesity-inducing crisps from environment-destroying plastic packets.*

*Just ate a grass-fed rib eye steak. This makes me #vegan by proxy. #ClimateHoax*

*How to make a Vegan roll: Push the rose-eating, veg worshipping, flower chomping snowflake down a mountain #vegansausageroll*

*These militant idiots are damaging the #vegan cause more than anyone else*

I think I’ll stay on twitter another week then f*ck it off. It’s only vegan c*nts on about Brexit

Finally, two of the largest communities in the network are dominated by discussion of the GVSR. Both of the GVSR-dominated communities display what we term a *defender effect* characterised by posts focused on defending the GVSR and veganism. The first community does so using ridicule and sarcasm in response to the criticism aimed at the GVSR and Veganuary. As such, it is labelled *GVSR–* because it contains crude, aggressive, and even violent language from both critics and defenders. For example, this community contains several users attempting to defend vegans and the GVSR by condemning the attackers’ arguments. Many of these users openly state that they are not vegan but that they would stand up for people’s right to eat what they want. Their tone matches the vitriol they perceive in the anti-GVSR/vegan rhetoric. Some also flag the extent to which right-wing British politicians, and specifically supporters of Brexit became offended by a vegan sausage roll, in comparison with other issues, as the primary locus of their criticism. For example,

*Has anyone compared the set of men outraged about the Greggs vegan roll and Brexit voters? They are surely the same people.*

*I’m definitely not vegan, but I don’t get the problem…some people in the UK are vegan, it’s their right to choose their diet, why shouldn’t a company like GreggsOfficial cater to them? There must be more pressing issues to get worked up about?*

The second GVSR community, *GVSR+*, is generally positive. Users post about their desire to try the GVSR, their surprise about how good it is, and support for the idea of large franchises offering vegan options. There are also users exhibiting the defender effect by making jokes about the criticism it is receiving. Many of these users are not vegans themselves, but rather enjoy making fun of the people upset about the GVSR and veganism. Overall, these posts are more lighthearted and less vitriolic than those found in GVSR-. For example,

*I seriously want to try a vegan sausage roll, and yes, I know this means the marketing team at GreggsOfficial has roped me in*

*Greggs vegan sausage rolls taste like food and communism*

### Audience separation

**Fig. 2:**
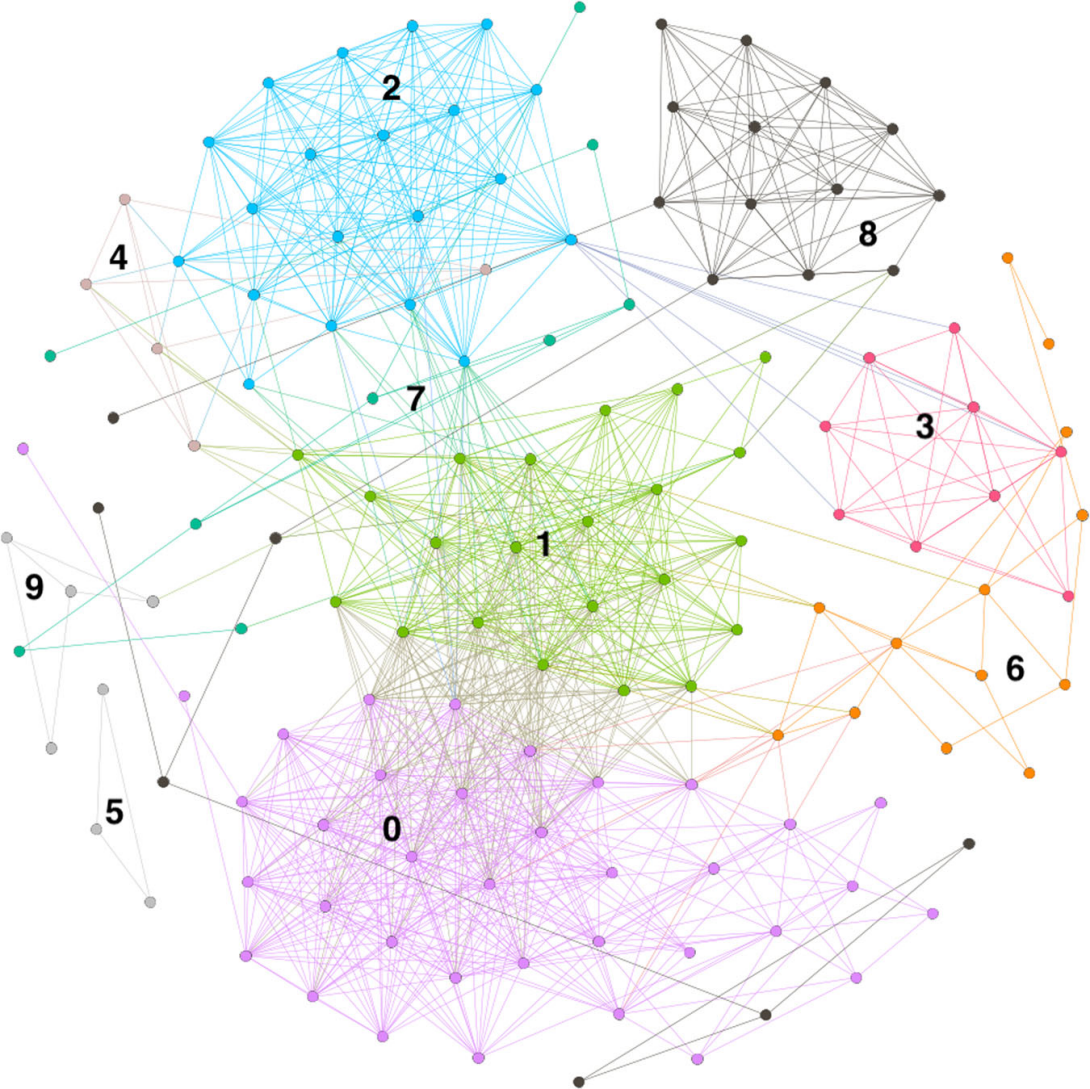
Veganuary 2019 projection network. Shows the network community structure of users with overlapping sets of retweeting users. Ten distinct communities emerge. They are labelled arbitrarily with digits. These digits match those found on the right hand side of Fig. 3.

Next, we discuss the results of the audience analysis. The monopartite graph, in which the most popular producer users are connected to one another if they have been retweeted by the same consumer users, contains 141 nodes and 996 edges. Figure 2 visualises the network.

**Fig. 3:**
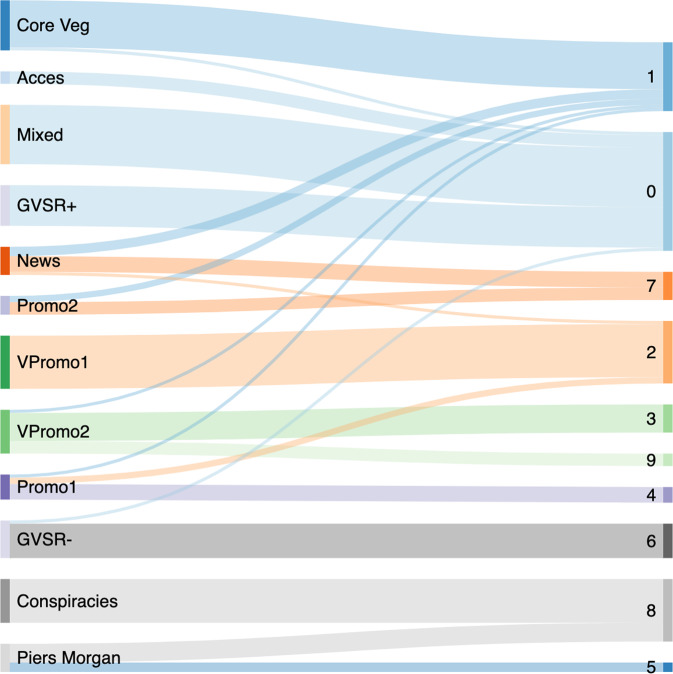
Flow chart to illustrate the composition of the communities in the projection network in terms of the retweet network communities. Retweet network communities listed on left. Projection network communities on right. Retweet network communities retain their labels while projection network communities are denoted with arbitrary digits. E.g., Community 1 in the projection network signifies that members of the following communities identified in the retweet network have overlapping audiences: Core Veganuary activist, News, Promo2, VPromo2, and Promo1.

Community detection to determine the extent to which the audiences of the communities in the discourse overlap, i.e., the relative magnitudes of which users tend to be retweeted by the same people, reveals ten communities. The modularity score of the network is 0.54. We do not undertake the same robustness checks here as we did in the retweet network because the size of the projection is much smaller and therefore less likely to be biassed (Shizuka & Farine, 2016). We do not attempt to label these communities the same way we did the retweet network communities as we are interested in determining what the community membership tells us about the audience structure of the underlying retweet network. To do so, we calculate the composition of these communities in terms of the retweet network communities, e.g., what communities the nodes in the projection communities represent in the retweet network. Figure 3 shows the results.

Overall, there is strong separation between all communities in the projection, with the exceptions of communities 1 and 0. Community 1 contains almost all of the core Veganuary support activists with four from the news community, two from a generic commercial community (*P2*), one from the other generic commercial community (*P1*), and one from one of the Veganuary-specific commercial community (*VP3*).

Meanwhile, community 0 combines all nodes from the Mixed community, the more supportive GVSR community (*GVSR+*), the antagonist access community, one user from the antagonist GVSR community (*GVSR–*), and one from the core Veganuary support community. These communities make up a majority of the overall retweet volume of the network, signifying that the audience shared by these users comprises a large proportion of the users active in the discourse. The fact that the content posted and shared by users in these communities centres on the GVSR, discussions on the accessibility of vegan diets, and jokes against vegans further emphasises the extent to which these narratives exceed the prevalence of the activist narratives in the discourse.

Critically, the projection shows that the audiences of most of the commercial communities remain separate from any other audiences in the discourse, therefore suggesting that commercial engagement with Veganuary 2019 failed to connect its audiences to the original activist discourse on the topic. The GVSR discourse is an exception, as its audience overlaps with other parts of the discourse, but this is due to how controversial and politicised the discourse surrounding the GVSR campaign became. In sum, the GVSR appears to be the exception which illustrates the rule, as the projection results show it was not the typical experience of commercial campaigns.

Interestingly, the more negative GVSR community does not share an audience with the other antagonist communities, but rather stays isolated, suggesting that the users most active with the negative elements of the GVSR discourse engaged an audience separate from the rest of the discourse. Meanwhile, the antagonist conspiracies community joins with most users from the antagonist Piers Morgan community to form one community in the projection, labelled 8, whereas the remaining users form their own community. These users are all supporters of the US-based far-right political conspiracy movement QAnon. They are connected to the Piers Morgan community in the retweet network but have their own separate audience distinct from the larger audience of the rest of the antagonist communities. It is possible that the politically right-wing and anti-vegan rhetoric of the Piers Morgan community appealed to these users and drew them into the discourse, thereafter further engaging their own particular audience of like-minded radicalised QAnon supporters. This is an example of one of the more serious and dangerous potential consequences of Morgan’s tirade against the GVSR veganism, which may have launched a domino effect beginning with broad attacks on liberal politics and ending with the engagement of radical groups.

## Discussion

### Contributions to understanding the impact of commercial engagement in Veganuary activist discourse

Our analyses show that commercial agents contributed lots of content and generated considerable interactions in the Veganuary discourse. However, the results of the audience projection indicates strong separation between their audiences and those of the activists, demonstrating that in general, commercial audiences in the discourse did not significantly overlap with those of the activist core. These results imply that regardless of true intention, the majority of commercial activity in Veganuary 2019 was limited to the insular engagement of isolated audiences that did not meaningfully contribute to increasing the reach of the activist movement. Commercial agents fall short of synergising their product promotion to the narratives and goals of the activist discourse. Their engagement serves to encourage consumption of their vegan products, but not engagement with the underlying movement.

An alternative interpretation of the projection results might be that commercial actors helped engage a new audience on the Veganuary topic, one that was not attained by activists. We are sceptical of this interpretation as it implies we know that this engagement with the commercial communities was as meaningful or informative as engagement with the activist community would have been, which we do not. Given the focus of the majority of commercial content on product promotion, and seemingly using the movement primarily as a marketing tool, and not explicitly on spreading awareness or information about the movement, as well as the example made by the GVSR campaign, we do not think this is likely.

This result is not surprising; it would be naive to assume that all corporations would want to do more than use the Veganuary campaign to promote their vegan products. While the Veganuary organisation frames part of their vision for corporate engagement with the campaign as just that, their vision for corporate involvement with the movement does not end there. They are not only interested in encouraging corporations to expand their vegan offerings and participate in the month-long campaign, but to also build the core vegan values of anti-cruelty and anti-exploitation into all their operations. As such, Veganuary as an organisation could do more to hold corporations accountable and work with them to ensure that when they engage with the campaign online, they do so in a way that actually benefits the movement, e.g., by building trust with sceptical consumers. As we see in several of the antagonist communities in the retweet network, perceptions of the vegan movement as insincere and associated with 'Big Veganism' can significantly damage efforts to raise awareness about how veganism can be a force for good. As a charitable organisation, Veganuary is in a good position to challenge corporate engagement to be more transparent about what ‘veganism’ really means to them. Doing so may separate corporations who are just in it for the marketing—and therefore should be excluded from the campaign—from those who actually share the Veganuary commitment.

### Contributions to understanding polarisation and contention in climate-diet discourse

The deep dive into the content of the tweets confirms the strong presence of polarisation and contention in the discourse. This is evidenced by the extent to which veganism was politicised in the discourse as well as the ways that commercial activity, specifically the GVSR, triggered severe antagonism, including the maligning of vegans as ‘liberal hippies’, and the drawing of parallels between standing *against* veganism with standing *for* various right-wing political initiatives like Brexit, Donald Trump, climate denialism, and QAnon. Moreover, the GVSR in conjunction with the EAT-*Lancet* report sparked many claims of veganism as a corporate and elitist hoax that further divided users. Although previous studies on climate-diet discourses provide reason to expect some polarisation and conflict in the Veganuary discourse, our observations are indicative of greater potential dangers for radicalisation if left unaddressed. Moreover, our work shows how distracting these elements can be from the core narratives of the activist movement.

Some might see these observations and say that all publicity is good publicity; although the discourse turned ugly in some areas, the fact that the GVSR led more people to talk about veganism is good for spreading awareness and bringing the discourse into the mainstream. We are sceptical of this argument. Activity in both GVSR communities did not often extend to vegan advocacy, but rather remained rooted in the GVSR controversy itself, politics, and trolling. While the backlash to the GVSR and veganism did subsequently bring in new users to defend veganism, we suspect these users joined due to underlying conflicts related to certain polarising political and social undercurrents, and not to engage in vegan activism itself. As such, we are not convinced that this defender effect outweighs the impact of the original backlash, which extended to highly politicised and radical attacks against vegans, not only the GVSR. Thus, it is clear that the GVSR campaign significantly affected the Veganuary 2019 discourse but in ways that were of more benefit to Greggs in terms of exposure and sales than support for the Veganuary movement.

Furthermore, the GVSR controversy highlights an additional concern: Given the way populist leaders and spokespeople normalise violations of the traditional moral order, e.g., racism, misogyny, and xenophobia (Bucy et al., 2020; Wodak, 2021; Wodak et al., 2021), in their ascent to power, there is a risk that narratives such as those launched by Piers Morgan against the GVSR and veganism will embolden the subscribers of antisocial ideologies, therefore further entrenching sources of societal discord. We suspect it is very likely that the engagement we observe in the discourse from users aligned with the right-wing of US and UK politics would not have happened without the intervention from Morgan. These users likely felt emboldened by the anti-vegan crusade initiated by Morgan and joined the fray, not because there is something inherently threatening about veganism, but rather because of how it is aligned with left-leaning political agendas. This outcome illustrates how online discussions of veganism and diet choice exemplify online ‘culture wars’.

To address this, scholars must critically reflect on why these discussions are such flashpoints of antagonism, and investigate the foundations of these reactions in order to identify ways of deconstructing them. Hank Rothgerber (2014 and with colleagues 2020) has begun investigating sources of aggression to veganism, and suggests that it partly stems from a dissonance between an omnivore’s dietary choices, and the perceived vegan message that these choices are cruel to animals and the environment. This dissonance—or ‘the Meat Paradox’ (Percival, 2022)—triggers responses of guilt and shame, which then lead to defensiveness. Determining a way to reform that reaction and/or address the underlying triggers may help resolve some of the conflict in discourses related to all kinds of vegan activism. However, psychological factors alone are not the only contributors to vegan antagonism. It is clear that additional kinds of cultural, social, and communication reforms are needed to neutralise the culture wars around veganism, specifically in relation to environmental protection. Future research should determine such pathways. It should be noted that the tenor of the culture war is not sustained by antagonistic voices alone; we also find evidence of vegan supporters who are indignant, righteous and sometimes even rhetorically violent against non-vegans. It is important to understand the motivations of these users and the net impact of their rhetoric on the discourse as well.

### Recommendations for improving commercial engagement with Veganuary activism

Recommendations for how to improve commercial engagement range a spectrum of options. The most straightforward might be to simply tag the Veganuary organisation’s account instead of just including the hashtag, as most did in the 2019 sample. Doing so would provide audience members with a direct link to a core source of information on the movement. Instead of clicking on the hashtag which directs the user to a selection of all posts containing it, many of which may not actually be informative about the movement, by clicking on the Veganuary account link, the user would see the organisation’s official profile with all of its own posts, links, and information on how to get involved.

A more involved option would entail companies explicitly coordinating their advertising efforts with those of the Veganuary organisation and of associated activists. For example, instead of competitions in which users retweeted a company’s post for a chance to win some vegan product, these competitions could require users to also engage with the Veganuary account, e.g., retweet one of their posts, register for the campaign, sign a statement of support, etc. This way commercial engagement would be guaranteed to better support the momentum of the activist movement by more directly associating its product promotion efforts with the campaign and by driving its audience to engage with it. Here the Veganuary organisation could deploy its members (or a bot—see the “Gender Pay Gap Bot” that replied to organisations celebrating International Women’s Day in 2022 with their current gender pay gap figures; Mellor, 2022) to directly challenge (or shame) corporations, especially those not formally aligned with them already, who make reference to the campaign in promotional tweets without full committing to vegan values. In doing so, the organisation would show to potential naysayers that it acknowledges the darker side of veganism, and put distance between it and the ethic the organisation hopes to promote. This approach would help build credibility for the organisation amongst those who perceive the vegan movement as beholden to impure corporate interests.

All potential improvements will need to contend with backlash from naysayers. It will therefore be critical for companies to take note of previous campaigns, such as the GVSR, and to develop strategies for navigating the challenges of engaging with the politicised landscape of veganism. This is a lesson that generalises beyond the Veganuary context to any discourse contending with climate-diet topics and debates.

### Limitations

Inevitably, our study has limitations. First, the analysis only pertains to the 2019 discourse. We focused on this year because of the EAT-*Lancet* report and the dramatic yet insightful event of the GVSR, but a natural extension of the work would be to examine the discourse in subsequent years tracing the arc of increased environmental activism, which began in late 2018, and continues to the present day with the Fridays for Future movement, and the coronavirus pandemic. The focus on Twitter is also limiting as the platform does not represent the global discourse on climate-diet issues. In addition, our method only considered retweets, which excludes the interaction signals that could be extracted from likes or passive consumption of content (i.e., a user can be affected by a tweet without interacting with it). Finally, the projection method for identifying audience overlap cannot tell us definitively about the sequence or causality of interactions. It can only tell us if such mutual interactions occurred. This is necessary to establish the existence (or absence) of mutual interaction, but it is not sufficient to determine the sequence in which the interactions may have occurred.

## Conclusion

This work shows that commercial engagement with Veganuary in 2019 fell short of connecting its audience to the vegan activist movement. The findings demonstrate that commercial entities do participate, but that this engagement is limited with respect to deepening public understanding of why veganism is important. In fact, in the eyes of many users, corporate engagement actively *detracts* from the perceived authenticity of the Veganuary movement. Given that one of the pillars of the Veganuary organisation’s strategy is to use corporate engagement to expand their reach, it is concerning that the participation of the companies that did engage with the movement on Twitter did not serve to facilitate interactions between their audiences and the Veganuary activists. Further research must therefore consider how engagement on social media from corporations who are at least ostensibly committed to the cause can meaningfully support its online activism.

Our results also emphasise the need to better understand the roots of vegan antagonism. We show that a significant volume of interactions and conflict in the discourse would not have occurred had the GVSR not touched on such a strong trigger point of political controversy. This phenomenon is not unique to vegan discourses on social media. It is instead emblematic of the culture war trend in which niche topics are politicised and turned into toxic and polarising public debates. Further research should endeavour to better understand the roots and dynamics of these debates, and to make them more civil and constructive.

## Supplementary information

Supplementary Materials

## Data Availability

Per the stipulations of the ethical review, the Twitter data itself cannot be made public because it contains information, which could be used to identify individuals who are not public figures. The data can be made available upon request for academic research purposes.

## Code availability

The code will be made available upon request.
