# Supplementary material for: Veganuary and the vegan sausage (t)rolls: conflict and commercial engagement in online climate-diet discourse
Source: Humanit Soc Sci Commun. 2022 Dec 19;9(1):455. doi: 10.1057/s41599-022-01464-2 (PMC9761638; doi:10.1057/s41599-022-01464-2)
Supplement: Supplementary file 1 — Supplementary Materials [file 41599_2022_1464_MOESM1_ESM.pdf]

Veganuary and the vegan sausage (t)rolls: conflict and commercial engagement in online climate-diet discourse

Mary Sanford* and Jamie Lorimer
*Corresponding author: mary.sanford@oii.ox.ac.uk

Supplementary Materials

1 Retweets as a signal of influence

A major focus of this work is to identify the extent to which the primary communities of the discourse overlap or diverge in terms of the audiences they influence, specifically those related to commercial agents. In order to do so, we must define how these audiences may be extracted from the data using one of the established operationalisations of the concept of influence on Twitter.

Twitter affords three primary modes of interaction to its users: following, mentioning, and retweeting. The *follower* network consists of unidirectional edges defined between individuals if one of them *follows* the other. A *mention* on Twitter occurs when one user includes the username of another user in their tweet or retweet. Each bears its own implications of a user's influence over others. For example, users with many followers may not necessarily carry the same influence potential as those who are mentioned or retweeted more frequently (Cha, Haddadi, Benevenuto, & Gummadi, 2010; Kwak, Lee, Park, & Moon, 2010). Many researchers have worked to determine which interaction provides the best indication of influence across the population of Twitter users. Most of this research uses the Merriam-Webster dictionary's definition of influence: "the power or capacity of causing an effect in indirect or intangible ways." As (Cha et al., 2010) admit, there is no concrete way to measure or define the force of influence, despite the many sociological theories on the topic. Instead, researchers of social media networks have accepted the practice of representing influence by the interactions between users as they discuss and share information.

In the early days of research on Twitter, scholars were tempted to believe that the more followers a user had, the larger their audience base, and therefore the more influence they could yield. Kwak et al. (2010) first set out to challenge this idea by comparing the impact of the number of followers, retweets, and mentions a user has on their potential to lead others to engage with a certain act and drive information spread. They find that the number of followers, or in-degree, provides a poor reflection of influence, mostly due to the fact that a user's followers does not reliably represent the full scope of the other users who might interact with them. Instead, they find the number of retweets, due to the function retweets serve to facilitate information diffusion to different audiences (Boyd, Golder, & Lotan, 2010), to be a better indication of a user's potential ability to engage others and mobilise action. The authors find that

mentions also correlate with retweets but they often do not bear the same intent nor information content as retweets, i.e. mentions are mostly meant to engage others in conversation instead of spreading information. They are therefore considered to have lower influence potential. Cha et al. (2010) carry out similar analyses and arrive at the same conclusion.

Relatedly, Teng, Pei, Morone, and Makse (2016) apply the formulation of collective influence presented in Morone and Makse (2015) to Twitter networks. Generally, they find that nodes with high collective influence are those who are responsible for the most diffusion of interactions across the network by virtue of their proximity to other nodes with many neighbours, or potential audience size. When applying this concept to Twitter, Teng et al. (2016) find that operationalising influence as retweets produces the best identification of users with the top diffusion scope and out-of-network audience reach over follower-count based metrics.

In the end, researchers have arrived at the consensus that retweets are the most reliable and straightforward indication of a user's influence over others. As described by Kwak et al. (2010),

> *"Retweeting in a social network can serve as a powerful tool to reinforce a message [...] Individual [Twitter] users have the power to dictate which information is important and should spread by the form of* ***retweet****, which collectively determines the importance of the original tweet. In a way we are witnessing the emergence of collective intelligence [via the retweet mechanism]."*

As such, retweets have become the standard approach to measuring influence on Twitter. Further studies suggest that beyond influence, retweet interactions can be indicative of ground truth alliances and opinion trends offline (Becatti, Caldarelli, Lambiotte, & Saracco, 2019; Cherepnalkoski & Mozetic, 2015; Cherepnalkoski & Mozetič, 2016; Grčar, Cherepnalkoski, Mozetič, & Kralj Novak, 2017; Laflin et al., 2013; Mastroeni, Naldi, & Vellucci, 2020; Tumasjan, Sprenger, Sandner, & Welpe, 2010). Furthermore, retweets do not only signify influence but also membership to a specific audience. The act of retweeting another user is explicit evidence of the consumption of that user's content. It may not always mean endorsement or support, but it does indicate that the retweeting user is part of the original user's larger audience of content consumers on the platform.

Thus, we are confident that the reliance on retweets in the present study builds on a well-established foundation of previous work in which retweets are presented as the key influence metric on Twitter.

One final point: we analyse the undirected connections between users. In doing so, we neglect the ways in which the status of users might affect their retweet count and instead focus on detecting potentially shared interests. Such undirected retweet networks have proven useful for identifying communities of users who share common views (Evkoski, Mozetič, Ljubešić, & Kralj Novak, 2020), which is the primary focus of this work. Undirected networks are less useful for providing additional measures of individual influence beyond retweet count, which rely on in- and out-degree ratios. As such measurements are not

necessary for our present research agenda, the undirected network is more appropriate.

2 Further details of the retweet network

2.1 Community detection with the Louvain algorithm

The Louvain community detection algorithm operates by iteratively optimizing the Newman-Girvan modularity of the network, meaning the clusters of users connecting more between each other than with users from other clusters (Newman, 2006). The process to determine this optimization consists of two steps. In the first step, changes in each community are made to optimize internal modularity. Then, a new network is built that consists of one node for each previously found community. The algorithm repeats the iterations until the first phase can make no further improvements in modularity. We perform 10 rounds of the community detection to ascertain the stability of the community allocation. Less than 0.1% of nodes change membership across the rounds so we conclude that the detection algorithm is stable.

2.2 Modularity and assortativty

Modularity is the ratio between the fraction of edges that fall between the network communities and the fraction we would expect if edges were distributed randomly (Brandes et al., 2008). Modularity is increased by edges falling within communities and lowered by edges falling between communities. This value reflects the extent to which communities in a given network are connected to one another. It can take on values between [-1/2, 1], with positive values closer to 1 indicating stronger separation between communities. This measure is routinely used as an approximation for the polarization of a network (Conover et al., 2011; Garimella & Weber, 2017; Porter, Mucha, Newman, & Friend, 2013; Porter, Onnela, & Mucha, 2009; Waugh, Pei, Fowler, Mucha, & Porter, 2009).

However, this measure is also known to be misleading for large networks and is not robust to several sampling biases (Shizuka & Farine, 2016). As such, we complement the modularity score with a measurement of the assortativity of the network based on the detected community structure. *Assortativity* is defined as a correlation coefficient that measures the association patterns between different types of nodes (Newman, 2002, 2003). As such, it takes on values between -1 and 1, where positive values indicate a stronger tendency for nodes to connect with nodes which are similar to them in some way, a phenomenon commonly referred to as *homophily* (McPherson, Smith-Lovin, & Cook, 2001), while negative values indicate the absence of this. Homophily has been found in several social networks including climate discourse on social media (Williams, McMurray, Kurz, & Hugo Lambert, 2015), and it is commonly utilized in investigations of echo chambers and polarization (Baumann, Lorenz-Spreen, Sokolov, & Starnini, 2020; Bessi et al., 2015; Blex & Yasseri, 2022; Cinelli, Morales, Galeazzi, Quattrociocchi, & Starnini, 2021; Karimi,

Génois, Wagner, Singer, & Strohmaier, 2018; Lee et al., 2019; Sayama, 2020; Singh, Sreenivasan, Szymanski, & Korniss, 2012). In the retweet network here, we diagnose the network assortativity in terms of the community identities provided by the community detection algorithm. In doing so, assortativity is not assessed based on some qualitative feature of the nodes but rather on their connections to other nodes, i.e. their interactions with certain kinds of information or narratives.

To ascertain the robustness of the modularity and assortativity measures, 100 random networks are simulated with the same number of nodes and edges as the retweet network, community detection is performed, and the resulting modularity and assortativity scores are calculated. The simulations generate a confidence interval for the scores in random graphs against which to compare the values observed in the Veganuary 2019 network. If the confidence interval does not contain the observed values, we can conclude that the observed values is not the result of chance and therefore indicates significant modularity and/or assortativity in the network. With this validation, the modularity and assortativity scores provide a compelling diagnosis of the extent to which the retweet network is divided into distinct communities of users engaging with similar topics and ideas.

2.3 Community annotation and labelling process

To begin the community annotation and labelling process, random samples of 200 tweets are taken from each community. The original dataset was collected without the intention to analyse tweet content and as such, the tweets have been truncated at 140 characters even though many extend beyond to an upper limit of 280 characters. Therefore, if the tweets in our samples extended beyond the truncation point, we retrieved the full text using either the Twitter Intelligence Tool or the Twitter Academic Research API. These samples were labelled at first focusing on whether the post is pro-Veganuary, anti-Veganuary, commercial, and/or related to news media. However, there is a need to be more specific in these annotations. First, there is a) both commercial promotion of vegan brands and products specifically tied to the Veganuary initiative, and b) commercial content unrelated to the initiative, e.g. promotions of vegan brands and products that run at all times of year. This happens most likely due to the inclusion of *vegan* in the search criteria which returns both content relevant to Veganuary, particularly the GVSR, but also many semi-false positives: content related to veganism in general but not related to Veganuary. This content is kept within the sample because it is still related to the discourse on veganism and as such, we want to know if the users promoting it interact with the core Veganuary discourse. Thus, we use Veganuary-commercial (*VPromo*) and generic-commercial (*Promo*) labels to differentiate the two types of commercial content. Within the pro-vegan activist content, there are posts defending veganism against the criticism it receives (labeled *Defence*) as well as some aggression from vegans against non-vegans and vegan critics (labelled *Vegan*

aggression). We also differentiate the explicitly pro-vegan activism (*Pro*) from other more random posts mentioning veganism (*Other*).

Moreover, there is a significant portion of posts dedicated solely to GVSR commentary and debate which often differs in nature and intent from other posts. As such, we differentiate the GVSR content from the other categories. These posts range from positive sentiment generally in support of the GVSR to more negative sentiment, either against the GVSR or defending the GVSR against its critics. We use the label *GVSR+* for more positive (in terms of valence) GVSR-focused tweets and *GVSR-* for more negative GVSR-focused tweets.

Once the labelling framework is finalised, we begin the annotations. For each tweet, we manually record which label best applies. Once all tweets per community have been labelled, we count how how many times each label occurs in the sample for each community. A community is then given the label with the highest score, i.e. the one that is most frequent among the sampled tweets of that community. Table S1 shows the results of this process. The antagonist communities vary in terms of focus, primarily in line with Piers Morgan against 1) veganism and the GVSR, 2) discussing vegan conspiracy theories, 3) complaining about the poor accessibility of vegan diets, and 4) denigrating vegans. As such, we further specify the prevailing focus of each of these communities in the label.

Table S1 Results of the labeling process for the 200 randomly selected tweets per community.

Comm Name	Pro	Vegan aggression	Vegan defense	Anti-vegan	GVSR-	GVSR+	Veganuary commercial	Generic commercial	News	Other
Core Support	157	0	0	0	4	4	16	11	1	7
VPromo1	4	0	3	1	1	5	113	66	4	3
VPromo2	7	0	0	1	3	5	102	73	2	7
GVSR+	10	2	6	6	65	78	3	13	1	16
GVSR-	11	2	9	12	109	10	12	19	6	10
Access/Trolls	45	2	20	83	4	1	1	15	0	29
Piers Morgan	4	5	4	85	13	16	6	5	29	33
Conspiracies	9	0	5	154	2	3	1	2	11	13
News	19	0	0	9	0	9	14	18	114	17
Promo 1	5	0	0	1	1	2	26	143	4	18
Promo 2	18	3	10	8	9	12	21	71	8	40
Mixed	33	15	26	11	4	7	28	16	0	60

Labels are listed in the first row. Communities are listed by name in the first column. *Core support* = primary Veganuary activism, *VPromo1/2* = commercial engagement explicitly tied to Veganuary, *GVSR+* = supportive/positive of GVSR, *GVSR-* = disparaging/insulting the GVSR, *Access/Trolls* = antagonist community focusing on low accessibility of veganism and denigrating vegans, *Piers Morgan* = antagonist community led by Piers Morgan and his attacks on the GVSR and vegans, *Conspiracies* = antagonist community espousing various malevolent conspiracies behind veganism, *Promo1/2* = commercial engagement not explicitly tied to Veganuary, *News* = community of news outlets sharing articles related to Veganuary/veganism, *Mixed* = community of random comments about veganism.

Table S2 Example tweets for each label.

Label	Example Tweet
Pro-Veganuary	Today marks 9 years #vegan! I never thought I could do it, but I did. 9 years later, hundreds of animals have been saved through my diet, and thousands more through my advocacy. If I could do it, so can you. http://eatingveg.org
Defense	Y'all are mad when animals go extinct but won't go vegan?
Vegan aggression	Oh look, four years later living on a plant-based diet and I'm still alive. IMAGINE THAT, f*ckers. #vegan
GVSR+	Not sure what people's problem is with @GreggsOfficial doing a vegan sausage roll. It's not replacing the regular one.
GVSR-	The f*ckwits that like to call you a "snowflake" are blockading a Gregg's in Manchester for selling vegan sausage rolls.
News	Which vegan milk is best for environment? @BBCScienceNews
Veganuary commercial	FOLLOW & RT to enter our #competition this #Veganuary You could #WIN this bundle of #Sriracha Mayo goodies! #FreebeeFriday
Generic commercial	Tomato Curry with Coconut Rice is #RecipeOfTheDay and it's glorious. It also happens to be easy, speedy and #vegan
Antagonist - Piers Morgan	Good Morning Britain - the vegan resistance starts today!
Antagonist - Access/Trolls	...Will this challenge be accompanied by Beyoncé and Jay Z vegan money...
Antagonist - Conspiracies	Why do processed food giant like Nestle/Kellogg/Pepsi support the push to make the world vegan (by EATLancet etc). Bc their foods ARE vegan: vast majority of cookies, crackers, chips etc in supermarkets are made from: wheat, corn, soy, vegetable oils, sugars and salt.
Other	Imagine having an all vegan family; is cocaine vegan

3 Further details of the projection

The method requires first building an undirected bipartite network between the primary content producers and consumers in the data set. A bipartite network defines interactions between two mutually exclusive sets of users. In this case, these groups are the the most popular users (those who received the most amount of retweets) per community and the users who retweet them.

A bipartite network contains two independent and disjoint sets of nodes, represented as $G = (U, V, E)$ where U and V are the sets of nodes and E is the set of edges. Sometimes these node sets are also referred to as the *layers* of the network. Moreover, edges can only exist between nodes of different layers, not between nodes of the same layer. In Becatti et al. (2019), the two layers of nodes are the content-producing and content-consuming users contained in their dataset. An edge exists between a content-producing user and a content-consuming user if the latter has retweeted the former. Retweets between producing users are not included, nor are those between consuming users.

Next, the bipartite network is projected onto the producing users layer to produce a weighted monopartite network of producing users, wherein the users share an edge if they have been retweeted by the same consuming users. Thus, two nodes in the projection are seen as equivalent not by the existence of an edge between them in the retweet network, but due to a strong overlap between their audiences.

Fig S1 depicts an example. The network on the left is a bipartite network with consuming users on the top layer and producing users on the bottom layer. Edges between users on the different layers signify that the consuming user has retweeted one or more posts of the producing user. For example, user a has retweeted user e, user b has retweeted users e, f, and g, and so on. The network on the right is the monopartite network which results from projecting the bipartite network onto the producing users layer. The edges here connect the users who have been retweeted by the same users, implying that they have a shared audience of users. In this example, users e, f, and g have been retweeted by the same users, while Users g and h share a different set of common retweeting users.

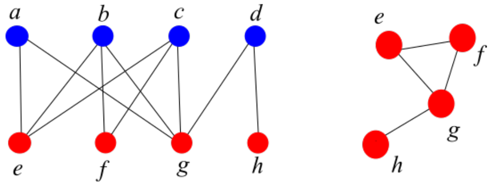

Fig. S1 Example bipartite network and resulting projection. The bipartite network is on the left: content-consuming users on the top layer, content-producing users on the bottom layer. The projection onto the content-producing users is shown on the right.

We define popular users as all those who received more than 1% of the sum total of retweets per community. We use retweets as an indicator of popularity and influence due to the established use of this variable for such purposes in the literature (as described in the S1). This approach preserves both globally and locally influential users. Thus, the projection is not biased towards globally popular users as it would be if were to take the top retweeted users over the full dataset. Doing so would obfuscate potentially important alliances between users in larger and smaller communities because those in smaller communities have a lower chance of being included in the top 1% of overall popular users.

Once the relevant users for each community have been identified, we extract the bipartite network containing all of their interactions with other users in the dataset, build the statistically-validated projection from this bipartite network, and carry out community detection on the resulting monopartite network. We take the same steps to validate these results as done for the retweet network. Next, the composition of each projection community in terms of the original retweet network communities is calculated. These results allow us to identify the common audiences shared by the users driving the discourse.

The projection method validates the resulting network by filtering out statistically insignificant edges using an entropy-based approach that prioritizes overlaps which cannot be explained by the raw number of tweets a user posts alone. This benchmark was initially presented in Saracco et al. (2017) and further refined in Bovet and Makse (2019); Bruno, Lambiotte, and Saracco (2022) for application to influence detection on retweet networks. More specifically, once the network has been projected to the selected layer, the algorithm

determines the statistical likelihood of an edge existing in a entropy-based null model with the same amount of nodes but variable number of edges, proceeding from an empty network to the fully connected one, via all possible link configurations. The null model maximizes the entropy of the system, constraining the degree sequence of the two layers in order to discount retweet contributions resulting only from the activity of the users. We do this to avoid favoring users who post a lot, therefore receiving potentially more retweets overall, but having less influential retweets than users who may post less often but receive more retweets per individual post. The result is a probability distribution over all edges in the network, which can be interpreted as the independent probability of existence per edge. Per Saracco et al. (2017), the p-value of each edge is then calculated using a Poisson distribution and only those below a given significance threshold are maintained to build the projection.

The bicm package for Python was written to implement the bipartite projection model for this purpose. We use the model's default presets to construct the projection, including a p-value value of 0.05 for the statistical testing of projected edges.

An alternative approach for selecting which nodes to use to build the projection would have been to take the same number of users per community, e.g. the top n most popular users. We also implemented this approach with various values of n but we could derive no criteria with which to rank or compare the resulting projections. As such, we decide that the most informative and sensible projection would be the one built from the same share of popularity per community, controlling for each community's contribution to retweet volume and therefore, aggregate influence. However, we compare the results of forming the projection with the top 10, 20, 30, 40, and 50 users per community and find the results to be robust.

Moreover, we use one final check of the projection results with 50 simulations using random samples of the same number of users per community as in the projection built according to the logic described above. These simulations resulted in only empty projections so we also tried doubling the number of nodes sampled per community. These additional simulations all also yielded empty projections. This happens because the projection is predicated on the presence of *V-motifs* in the bipartite network of producing users and consuming users. A V-motif connects users on the consuming side via a mutual connection to the producing side. An empty projection tells us that none of the V-motifs found in the bipartite network, if there are any, are statistically likely to be present beyond random chance. As such, we conclude that the results of the original projection identify statistically significant audiences within the sample and the information production-consumption relations in the discourse.

References

Baumann, F., Lorenz-Spreen, P., Sokolov, I.M., Starnini, M. (2020). Modeling echo chambers and polarization dynamics in social networks. *Physical*

Review Letters, *124*(4).

https://doi.org/10.1103/PhysRevLett.124.048301

Becatti, C., Caldarelli, G., Lambiotte, R., Saracco, F. (2019). Extracting significant signal of news consumption from social networks: the case of twitter in italian political elections. *Palgrave Communications*, *5*, 1–16.

https://doi.org/10.1057/s41599-019-0300-3

Bessi, A., Coletto, M., Davidescu, G.A., Scala, A., Caldarelli, G., Quattrociocchi, W. (2015). Science vs conspiracy: Collective narratives in the age of misinformation. *PLOS ONE*, *10*(2).

https://doi.org/10.1371/journal.pone.0118093

Blex, C., & Yasseri, T. (2022). Positive algorithmic bias cannot stop fragmentation in homophilic networks. *The Journal of Mathematical Sociology*, *46*(1), 80-97.

https://doi.org/10.1080/0022250X.2020.1818078

Bovet, A., & Makse, H.A. (2019). Influence of fake news in twitter during the 2016 us presidential election. *Nature Communications*, *10*(1), 1–14.

https://doi.org/10.1038/s41467-018-07761-2

Boyd, D., Golder, S., Lotan, G. (2010). Tweet, tweet, retweet: Conversational aspects of retweeting on twitter. *2010 43rd hawaii international conference on system sciences* (pp. 1–10). Honolulu, HI: IEEE. https://doi.org/10.1109/HICSS.2010.412

Brandes, U., Delling, D., Gaertler, M., Gorke, R., Hoefer, M., Nikoloski, Z., Wagner, D. (2008). On modularity clustering. *IEEE Transactions on Knowledge and Data Engineering*, *20*(2), 172–188.

https://doi.org/10.1109/TKDE.2007.190689

Bruno, M., Lambiotte, R., Saracco, F. (2022). Brexit and bots: characterizing the behaviour of automated accounts on twitter during the uk election. *EPJ Data Science*, *11*(1), 1–24.

https://doi.org/10.1140/epjds/s13688-022-00330-0

Cha, M., Haddadi, H., Benevenuto, F., Gummadi, K.P. (2010). Measuring user influence in twitter: The million follower fallacy. *Icwsm '10: Proceedings of international aaai conference on weblogs and social.*

Cherepnalkoski, D., & Mozetic, I. (2015). A retweet network analysis of the european parliament. *2015 11th international conference on signal-image technology internet-based systems (sitis)* (pp. 350–357). https://doi.org/10.1109/SITIS.2015.8

Cherepnalkoski, D., & Mozetič, I. (2016). Retweet networks of the european parliament: evaluation of the community structure. *Applied Network Science*, *1*(1), 1–20.

https://doi.org/10.1007/s41109-016-0001-4

Cinelli, M., Morales, G.D.F., Galeazzi, A., Quattrociocchi, W., Starnini, M. (2021). The echo chamber effect on social media. *Proceedings of the National Academy of Sciences*, *118*(9).

https://doi.org/10.1073/pnas.2023301118

Conover, M., Ratkiewicz, J., Francisco, M., Goncalves, B., Menczer, F., Flammini, A. (2011). Political polarization on twitter. *Proceedings of the International AAAI Conference on Web and Social Media*, *5*(1), 89–96. Retrieved from https://ojs.aaai.org/index.php/ICWSM/article/view/14126

Evkoski, B., Mozetič, I., Ljubešić, N., Kralj Novak, P. (2020). *A slovenian retweet network 2018-2020.*

Garimella, V.R.K., & Weber, I. (2017). A long-term analysis of polarization on twitter. *Aaai conference on web and social media.*

Grčar, M., Cherepnalkoski, D., Mozetič, I., Kralj Novak, P. (2017). Stance and influence of twitter users regarding the brexit referendum. *Computational Social Networks*, *4*(1).

https://doi.org/10.1186/s40649-017-0042-6

Karimi, F., Génois, M., Wagner, C., Singer, P., Strohmaier, M. (2018). Homophily influences ranking of minorities in social networks. *Scientific reports*, *8*(1), 1–12.

https://doi.org/10.1038/s41598-018-29405-7

Kwak, H., Lee, C., Park, H., Moon, S. (2010). What is twitter, a social network or a news media? *Proceedings of the 19th international conference on world wide web* (pp. 591–600). Association for Computing Machinery. https://doi.org/10.1145/1772690.1772751

Laflin, P., Mantzaris, A.V., Ainley, F., Otley, A., Grindrod, P., Higham, D.J. (2013). Discovering and validating influence in a dynamic online social network. *Social Network Analysis and Mining*, *3*(4), 1311–1323.

https://doi.org/10.1007/s13278-013-0143-7

Lee, E., Karimi, F., Wagner, C., Jo, H.-H., Strohmaier, M., Galesic, M. (2019). Homophily and minority-group size explain perception biases in social networks. *Nature Human Behaviour*, *3*(10), 1078–1087.

https://doi.org/10.1038/s41562-019-0677-4

Mastroeni, L., Naldi, M., Vellucci, P. (2020). Calibration of an agent-based model for opinion formation through a retweet social network. *Ceur workshop proceedings* (p. 161-173).

McPherson, M., Smith-Lovin, L., Cook, J.M. (2001). Birds of a feather: Homophily in social networks. *Annual Review of Sociology*, *27*(1), 415–444.

https://doi.org/10.1146/annurev.soc.27.1.415

Morone, F., & Makse, H.A. (2015). Influence maximization in complex networks through optimal percolation. *Nature*, *524*(7563), 65–68.

https://doi.org/10.1038/nature14604

Newman, M.E.J. (2002). Assortative mixing in networks. *Physical Review Letters*, *89*(20).

https://doi.org/10.1103/PhysRevLett.89.208701

Newman, M.E.J. (2003). Mixing patterns in networks. *Physical Review E*, *67*(2).

https://doi.org/10.1103/PhysRevE.67.026126

Newman, M.E.J. (2006). Modularity and community structure in networks. *Proceedings of the National Academy of Sciences*, *103*(23).

Porter, M.A., Mucha, P.J., Newman, M., Friend, A.J. (2013). *Community structure in the united states house of representatives* (SSRN Scholarly Paper). Rochester, NY: Social Science Research Network.

Porter, M.A., Onnela, J.-P., Mucha, P.J. (2009). Communities in networks. *arXiv:0902.3788 [cond-mat, physics:nlin, physics:physics, stat]*.

Saracco, F., Straka, M.J., Clemente, R.D., Gabrielli, A., Caldarelli, G., Squartini, T. (2017). Inferring monopartite projections of bipartite networks: an entropy-based approach. *New Journal of Physics*, *19*(5).

https://doi.org/10.1088/1367-2630/aa6b38

Sayama, H. (2020). Enhanced ability of information gathering may intensify disagreement among groups. *arXiv:2002.01049 [nlin, physics:physics, q-bio]*.

Shizuka, D., & Farine, D.R. (2016). Measuring the robustness of network community structure using assortativity. *Animal Behaviour*, *112*, 237–246.

https://doi.org/10.1016/j.anbehav.2015.12.007

Singh, P., Sreenivasan, S., Szymanski, B.K., Korniss, G. (2012). Accelerating consensus on coevolving networks: The effect of committed individuals. *Physical Review E*, *85*(4).

https://doi.org/10.1103/PhysRevE.85.046104

Teng, X., Pei, S., Morone, F., Makse, H.A. (2016). Collective influence of multiple spreaders evaluated by tracing real information flow in large-scale social networks. *Scientific Reports*, *6*(1).

https://doi.org/10.1038/srep36043

Tumasjan, A., Sprenger, T., Sandner, P., Welpe, I. (2010). Predicting elections with twitter: What 140 characters reveal about political sentiment. *Proceedings of the International AAAI Conference on Web and Social Media*, *4*(1).

Waugh, A.S., Pei, L., Fowler, J.H., Mucha, P.J., Porter, M.A. (2009). *Party polarization in congress: A network science approach* (SSRN Scholarly Paper). Rochester, NY: Social Science Research Network.

Williams, H.T.P., McMurray, J.R., Kurz, T., Hugo Lambert, F. (2015). Network analysis reveals open forums and echo chambers in social media discussions of climate change. *Global Environmental Change*, *32*, 126–138.

https://doi.org/10.1016/j.gloenvcha.2015.03.006
